# Demographics and Characteristics of Acutely Decompensated Heart Failure (ADHF) Patients in a Tertiary Care Center in Saudi Arabia

**DOI:** 10.7759/cureus.35724

**Published:** 2023-03-03

**Authors:** Hassan Alalawi, Hamza L Fida, Omar A Bokhary, Majed A Alhuzali, Abdullah F Alharbi, Faisal Y Alhodian, Mohammed R Alsahari, Aisha M Siddiqui

**Affiliations:** 1 Medicine and Surgery, King Abdulaziz University Faculty of Medicine, Jeddah, SAU; 2 Internal Medicine, King Abdulaziz University Hospital, Jeddah, SAU

**Keywords:** heart failure, adhf, heart disease, ejection fraction, acute decompensated heart failure

## Abstract

Background: Acute decompensated heart failure (ADHF) has been defined as the gradual or rapid change in heart failure (HF) signs and symptoms resulting in a need for urgent therapy. Patients with ADHF usually have multiple comorbidities that contribute to the severity of exacerbation at admission, including diabetes mellitus and chronic kidney disease. The prognosis for these individuals is generally poor, with a high risk of readmission and death after discharge. Unfortunately, there are limited studies in Saudi Arabia reporting the characteristics of such patients. We aim to study the demographics and characteristics of ADHF patients admitted to King Abdulaziz University Hospital (KAUH) and analyze gender discrepancies and in-hospital mortality.

Methods: This retrospective record review was conducted at KAUH. The study included 425 patients diagnosed with ADHF. The New York Heart Association (NYHA) classification, underlying etiology of HF, comorbidities, left ventricular ejection fraction (LVEF), vital signs, comprehensive metabolic panel at admission, and in-hospital mortality were evaluated and analyzed.

Results: The majority of the patients were males (52.5%), and the average age was 63 ± 13.75 years. The most prevalent etiology of HF was hypertensive heart disease (51.8%), followed by ischemic heart disease (42.8%). The most common comorbidity was diabetes mellitus (73.6%), and the most common medication administered was diuretics (95.5%). The mean LVEF was 37.9% ± 16.0. In-hospital mortality occurred in 10.4% of patients. The mean length of hospitalization was 16.7 ± 86.2 days. The mean blood urea nitrogen (BUN) (17.18 ± 11.16) and creatinine (243.34 ± 222.27) were higher in patients with in-hospital mortality. The mean difference was statistically significant (P = 0.003 and P = 0.014). A higher length of hospitalization was significantly associated with in-hospital mortality (P = 0.036).

Conclusion: We found more than half of our sample to be males and diabetes mellitus to be common among ADHF patients. Elevated BUN and creatinine levels at the time of presentation, as well as patients who had been in the hospital for a more extended period of time, were found to be associated with an increased risk of in-hospital mortality.

## Introduction

According to the recent universal definition of heart failure (HF) proposed by the Heart Failure Society of America, Heart Failure Association of the European Society of Cardiology, and Japanese Heart Failure Society, "HF is a clinical syndrome with current or prior symptoms and/or signs (including breathlessness, orthopnea, ankle swelling, elevated jugular venous pressure, and third heart sound, among others) caused by a structural and/or functional cardiac abnormality (as determined by ejection fraction (EF) <50%, abnormal cardiac chamber enlargement, E/E′ >15, moderate/severe ventricular hypertrophy, or moderate/severe valvular obstructive or regurgitant lesion) and corroborated by at least one of the following: elevated natriuretic peptide levels and/or objective evidence of cardiogenic pulmonary or systemic congestion by diagnostic modalities such as imaging (e.g. by chest X-ray or elevated filling pressures by echocardiography) or hemodynamic measurement (e.g. right heart catheterization and pulmonary artery catheter) at rest or with provocation (e.g. exercise)" [1]. More than 23 million people worldwide are affected by HF [2], while approximately 320,000 patients are affected by HF in Saudi Arabia [3]. Worsening of preexisting HF is termed acute decompensated heart failure (ADHF) [4]. ADHF has been defined as "gradual or rapid change in heart failure signs and symptoms resulting in a need for urgent therapy" [5]. Patients present with signs and symptoms of fluid overload and congestion, including exertional dyspnea, orthopnea, S3 gallop, elevated jugular venous pressure, and peripheral edema [4]. Patients with ADHF usually have multiple comorbidities that contribute to the severity of admission, including diabetes mellitus and chronic kidney disease [4,6]. The prognosis for these individuals is generally poor, with a high risk of readmission and death after discharge [4,7]. There are multiple identified conditions that can precipitate HF, leading to decompensation and subsequent hospital admission. These include arrhythmias, uncontrolled hypertension, renal insufficiency, diabetes mellitus, infection, and non-compliance with the treatment regimen [8-12].

Previous studies in Saudi Arabia reporting the demographics and characteristics of HF patients have been published. A retrospective study in Riyadh reported that most patients were males and that hypertension was the predominant comorbid illness, followed by diabetes mellitus [13]. Another study that included patients from four centers in Riyadh and Buraida similarly reported that the majority of patients were males and that hypertension and diabetes mellitus were the most frequent comorbidities [14]. Moreover, in a retrospective cohort study on HF patients in the Eastern region, the majority of HF patients were males [15]. The most common comorbidities were hypertension and diabetes mellitus in the three studies mentioned [13-15]. The previously mentioned studies reported demographic, clinical, echocardiographic, and therapeutic characteristics of chronic HF patients admitted to wards or visiting outpatient clinics. However, there are limited studies in Saudi Arabia reporting the characteristics of ADHF patients admitted to the hospital. Thus, we aim to study the demographics and characteristics of ADHF patients admitted to King Abdulaziz University Hospital (KAUH) and analyze the gender discrepancy and rate of in-hospital mortality.

## Materials and methods

Study setting and participants

A retrospective record review was conducted at KAUH, a tertiary care center in Jeddah, Saudi Arabia. This study was approved by the KAUH Institutional Review Board (reference number: 136-22). The procedures followed were in accordance with the responsible committee's ethical standards based on the Good Clinical Practice guidelines. As the study was conducted retrospectively, informed consent was waived.

**Figure 1:**
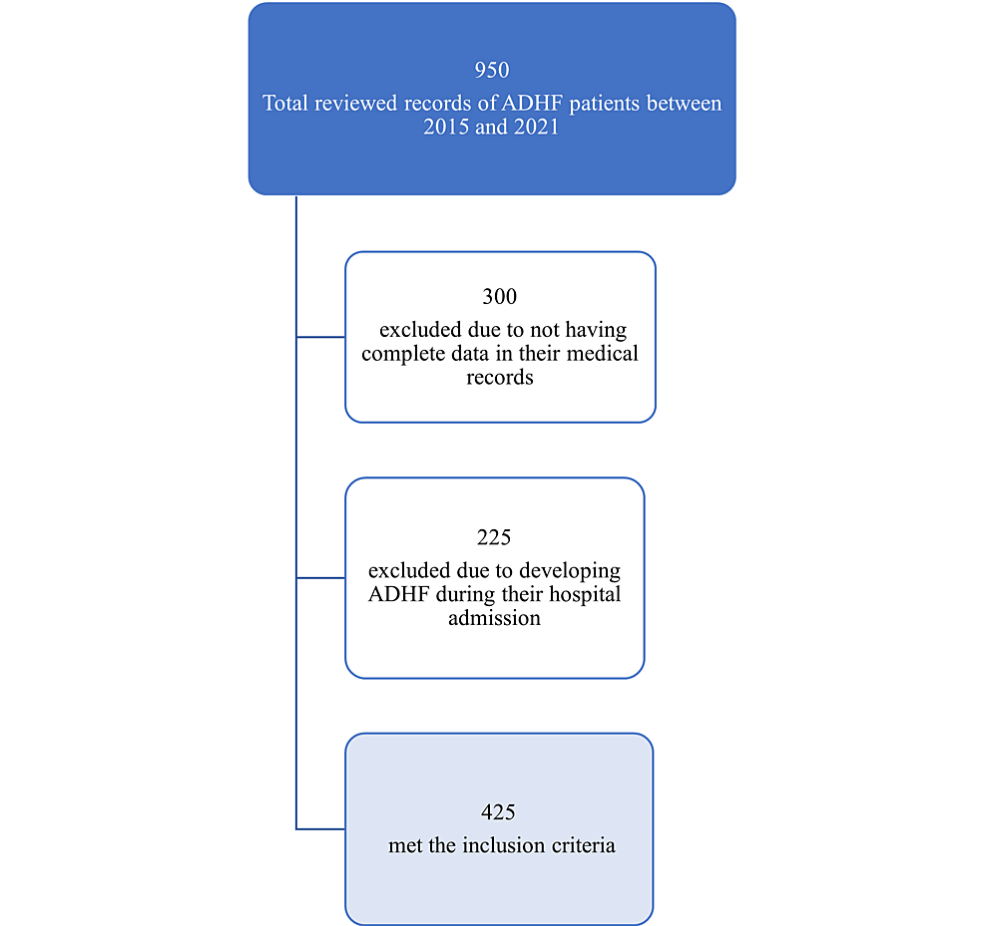
Flowchart of medical records review ADHF: acute decompensated heart failure.

The diagnosis of HF was established based on the clinical presentation of the patient and echocardiographic findings. We collected and analyzed the data of 950 adult patients (>18 years) diagnosed with ADHF at the time of presentation to the hospital between 2015 and 2021. The number of patients who fulfilled the inclusion criteria was 425. Patients who developed ADHF during admission or with incomplete data records were excluded (Figure 1).

Evaluated data

Data extracted from the electronic medical records included age, gender, nationality, date of admission, date of discharge or death, New York Heart Association (NYHA) classification, underlying etiology of HF, comorbidities, left ventricular ejection fraction (LVEF), smoking status, administered medications during hospitalization, select vital signs at admission (oxygen saturation, blood pressure, and heart rate), body mass index (BMI), brain natriuretic peptide (BNP), hemoglobin creatinine, blood urea nitrogen (BUN), sodium, potassium, random glucose, glycosylated hemoglobin (HbA1C), total cholesterol, high-density lipoprotein (HDL) cholesterol, low-density lipoprotein (LDL) cholesterol, triglycerides, and albumin. In-hospital mortality and length of hospitalization were also collected.

Statistical analysis

Data entry was performed by using Microsoft Excel 2016 (Microsoft Corporation, Redmond, WA). ‏Statistical Package for the Social Sciences (SPSS) for Windows (version 21; IBM Corp., Armonk, NY) was used for statistical analysis. Mean and standard deviation (SD) were calculated to describe continuous variables while numbers and percentages were used for categorical variables. The chi-square test, independent t-test, and ANOVA test were used to evaluate the differences between qualitative and continuous variables. Pearson’s correlation test was used to evaluate relationships between continuous variables. A p-value < 0.05 was considered significant.

## Results

A total of 425 patients met our inclusion criteria and were included in this study. Over half of our sample were males (n = 223, 52.5%), the average age was 63 ± 13.75 years, and the average BMI was 30.1 ± 7.6. Detailed patient characteristics and gender associations are outlined in Table 1. The most prevalent etiology of HF in our sample was hypertensive heart disease (n = 220, 51.8%), followed by ischemic heart disease (n = 182, 42.8%). Other frequent comorbidities in this study population were diabetes mellitus (n = 313, 73.6%) and anemia (n = 201, 47.3%).

**Table 1:** Demographics and characteristics * Statistically significant. PTCA: percutaneous transluminal coronary angioplasty; CABG: coronary artery bypass grafting; TIA: transient ischemic attack.

Variable	Total	Male	Female	P-value	
Sample size	425	223 (52.5%)	202 (47.5%)	-	
Age (years)	63 ± 13.75	61.0 ± 13.4	65.2 ± 13.7	0.001*	
Smoker	92 (21.6%)	56 (83.6%)	11 (16.4%)	<0.001*	
BMI	30.1 ± 7.6	29.3 ± 7.1	30.9 ± 8.0	0.033*	
Etiologies					
Ischemic heart disease	182 (42.8%)	115 (63.2%)	67 (36.8%)	<0.001*	
Hypertensive heart disease	220 (51.8%)	103 (46.8%)	117 (54.2%)	0.020*	
Dilated cardiomyopathy	23 (5.4%)	17 (73.9%)	6 (26.1%)	0.057	
Valvular heart disease	62 (14.6%)	24 (38.7%)	38 (61.3%)	0.027*	
Atrial fibrillation	58 (13.6%)	23 (39.7%)	35 (60.3%)	0.05	
PTCA or CABG	28 (6.6%)	23 (82.1%)	5 (17.9%)	0.002*	
Infection	96 (30.1%)	50 (52.1%)	46 (47.9%)	0.855	
Non-compliance	67 (21.0%)	43 (64.2%)	24 (35.8%)	0.020*	
Stroke or TIA	33 (7.8%)	11 (33.3%)	22 (66.7%)	0.035*	
Comorbidities					
Diabetes mellitus	313 (73.6%)	166 (53.0%)	147 (47.0%)	0.780	
Hypertension	342 (80.5%)	172 (50.3%)	170 (49.7%)	0.089	
Autoimmune disease	10 (2.4%)	3 (30.0%)	7 (70.0%)	0.263	
Thyroid disease	48 (11.3%)	16 (33.3%)	32 (66.7%)	0.008*	
Chronic liver disease	17 (4.0%)	8 (47.1%)	9 (52.9%)	0.835	
Chronic kidney disease	94 (22.1%)	45 (47.9%)	49 (52.1%)	0.371	
Respiratory disease	88 (20.7%)	37 (42.0%)	51 (58.1%)	0.038*	
Malignancy	24 (5.6%)	10 (41.7%)	14 (58.3%)	0.378	
Cerebrovascular disease	33 (7.8%)	16 (48.5%)	17 (51.5%)	0.767	
Anemia	201 (47.3%)	97 (48.3%)	104 (51.7%)	0.121	
Left ventricular ejection fraction					
Normal (>=55%)	71 (21.0%)	21 (29.6%)	50 (70.4%)	<0.001*	
Borderline low (50-54%)	37 (10.9%)	22 (59.5%)	15 (40.5%)		
Impaired (36-49%)	56 (16.6%)	28 (50.0%)	28 (50.0%)		
Severely impaired (<= 35%)	174 (51.5%)	119 (68.4%)	55 (31.6%)		

Out of the 182 patients with ischemic heart disease as the underlying etiology, 115 (63.2%) were males compared to 67 (36.8%) female patients. This difference was statistically significant (P < 0.001). In contrast, female patients (n = 117, 53.2%) were statistically more likely to have hypertensive heart disease as the underlying cause than male patients (n = 103, 46.8%) (P = 0.02). The mean BMI was 29.3 ± 7.16 in male patients compared to 30.89 ± 8.04 in female patients (P = 0.033).

**Figure 2:**
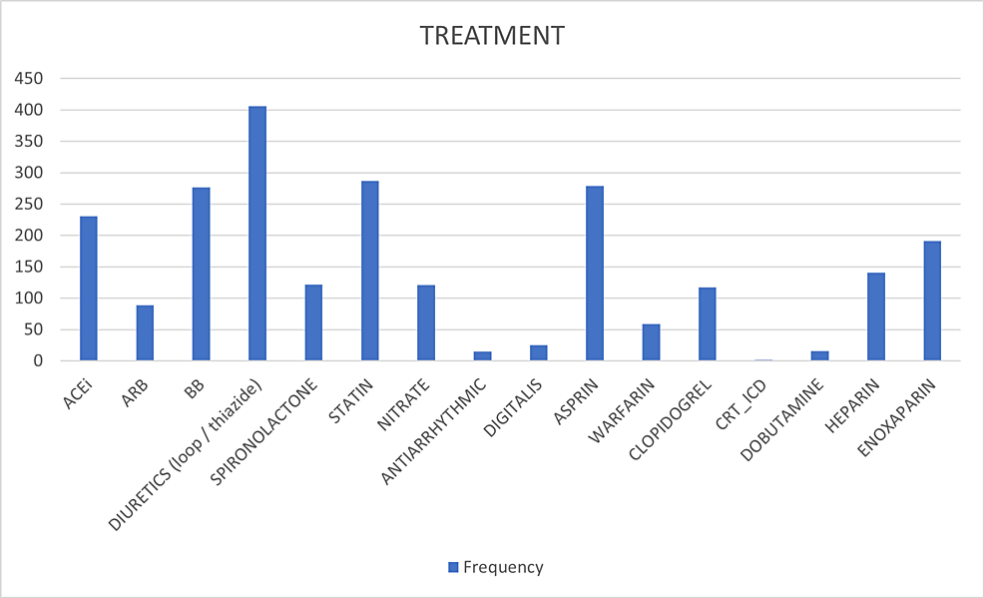
Medications prescribed at the time of admission ACEi: angiotensin-converting enzyme inhibitors; ARB: angiotensin receptor blockers; BB: beta blockers; CRT-ICD: cardiac resynchronization therapy-implantable cardioverter defibrillator.

Significant variations in this sample were observed in vital sign readings at triage, as shown by large standard deviations in oxygen saturations and systolic blood pressures recorded in Table 2. On the other hand, consistently abnormal laboratory values were hemoglobin A1C (mean = 8.03% ± 2.19) and serum high-density lipoprotein (mean = 0.1 mmol/L ± 0.38). Regarding inpatient treatment, the most common medication class administered was diuretics (loop or thiazide diuretics; n = 406, 95.5%), followed by statins (n = 287, 67.5%) (Figure 2).

**Table 2:** Vitals and labs at the time of admission HbA1c: glycosylated hemoglobin; HDL: high-density lipoprotein; LDL: low-density lipoprotein; NT‐proBNP: N-terminal pro-B-type natriuretic peptide.

Variable	Mean ± SD
Admission vital signs	
Oxygen saturation (%)	94.9 ± 8.53
Systolic blood pressure (mmHg)	132.9 ± 24.5
Diastolic blood pressure (mmHg)	74.9 ± 15
Heart rate (bpm)	88.3 ± 17.8
Echocardiography	
Left ventricular ejection fraction	37.9 ± 16
Laboratory investigations	
Hemoglobin (g/dL)	11.7 ± 7
Serum creatinine (μmol/L)	164.8 ± 145.2
Blood urea nitrogen (mmol/L)	12.3 ± 14.6
Sodium (mEq/L)	134.3 ± 8
Potassium (mEq/L)	4.46 ± 6.3
Glucose (mmol/L)	10.3 ± 5.2
HbA1c (%)	8.03 ± 2.2
Total cholesterol (mmol/L)	3.53 ± 1.3
HDL (mmol/L)	0.10 ± 0.3
LDL (mmol/L)	3.70 ± 11.7
Triglycerides (mmol/L)	1.38 ± 0.8
Albumin (g/dL)	30.1 ± 6.4
NT‐proBNP (pmol/l)	12175.5 ± 20731

Left ventricular ejection fraction

Considerable variation in LVEF was observed (37.9 ± 16.0). A positive correlation between LVEF and BMI was found (r = 0.149), which was statistically significant (P = 0.007). This finding was further supported by the ANOVA test, which showed a statistically significant difference in mean BMI between different LVEF groups (P = 0.041). Likewise, LVEF positively correlated with age (r = 0.215) with a statistically significant relationship (P < 0.001). The mean LVEF in male patients was 33.10 ± 14.23, while the mean LVEF in female patients was 43.55 ± 16.17. The difference between the means was statistically significant (P < 0.001).

Hospital outcomes

The average levels of BUN (17.18 ± 11.16) and creatinine (243.34 ± 222.27) were higher in patients who died compared to those who did not (11.78 ± 14.88 and 155.65 ± 130.88, respectively). The difference in means was statistically significant (P = 0.003 and P = 0.014, respectively). In addition, the mean BNP level was higher in patients who suffered in-hospital mortality (26799.23 ± 39253.36; P = 0.033).

In-hospital mortality occurred in 44 (10.4%) patients. The mean length of hospitalization was 16.7 ± 86.27 days. Patients who died had a higher mean length of hospitalization (91.66 ± 256.62) than patients who did not die (8.05 ± 11.29) (P = 0.036).

## Discussion

In our study, over half of our sample was found to be males (52.5%) and the most prevalent comorbidity was hypertension followed by diabetes. The mean age of our sample was 63 ± 13.75 years. Our results are in line with other research studying HF conducted in the literature. A study that included patients from four centers in Riyadh and Buraida similarly reported that the majority of patients were males and that hypertension and diabetes mellitus were the most frequent comorbidities. The mean age reported was 55.6 years [14]. Moreover, in a retrospective cohort study on HF patients in the Eastern region of Saudi Arabia, the majority of HF patients were males, and the mean age was 63 years. The most common comorbidities were hypertension and diabetes mellitus as well [15]. A variety of factors could contribute to this result but mainly it could be explained by the fact that males generally are more prone to cardiac disease as the cardioprotective effect estrogen has on females is well understood. Furthermore, females have fewer comorbidities in our sample and also less number of smokers; thus, females are less likely to have cardiac diseases leading to HF.

In our study, a significant relationship was observed between medication non-compliance and the male gender (P = 0.02). This was also followed in multiple papers, but overall, gender inconsistency in this matter exists. Some studies found that women were more compliant than men [16,17], while others found contradicting data [18,19]. It is, therefore, imperative to understand what may affect the compliance level in our community. It can be hypothesized that patients with a lower literacy level and economic status could have lower compliance. On the other hand, the prescribing physician and the pharmacist play a crucial role in ensuring that the patient understands how this affects their disease course. We strongly encourage physicians to focus on educating their patients and those around them on the importance of compliance.

LVEF is an essential indicator of cardiac health, as a lower LVEF is associated with an increased risk of HF and other cardiovascular diseases. Our research found a positive correlation between LVEF and BMI (r = 0.149), which was statistically significant (P = 0.007). Studies have demonstrated a positive correlation between LVEF and BMI [20,21]. Specifically, a paper published in the International Journal of Cardiology found that higher BMI was associated with a lower LVEF, even after adjusting for other risk factors such as age, gender, and lifestyle factors [20]. Similarly, another study found that a higher BMI was associated with a lower LVEF [21]. These findings suggest that maintaining a healthy weight may be beneficial for maintaining a healthy heart.

Another significant association between the male sex and low LVEF (P < 0.001) was found. A paper published in 2023 by Zhang et al. partially agrees with our result as they also concluded that males had statistically significant low LVEF in their sample [22]. However, contrary to their result, where males in their sample had hypertension as the etiology, our sample showed hypertension as the most prevalent etiology (51.8%) but it was higher (54.2%) and statistically significant (P = 0.02) in females rather than males. It is interesting to have this variation in both findings that could be due to the differences in sample sizes and our sample's apparent differences in ethnicity. The study result indicates that males are more likely to have a worse ejection fraction.

Studies have consistently demonstrated that smoking prevalence is higher among men than women in most countries. For example, a review of smoking prevalence in Europe revealed that there were more male smokers than female smokers [23]. Similarly, the Centers for Disease Control and Prevention (CDC) reported that smoking prevalence was significantly higher among men than women in the United States [24]. This gender-based difference in smoking prevalence is likely due to differences in social norms, access to cigarettes, and marketing strategies targeted toward men [24]. This finding is further supported by the results of this study, which showed that male patients (83.6%) were more likely to be smokers compared to female patients (16.4%), and this difference was statistically significant (P = 0.001).

Ischemic heart disease (IHD) is a significant cause of morbidity and mortality worldwide, with a clear gender-based difference in prevalence. In this study, 42.8% of the participants had IHD. Of these, 63.2% were male, and only 36.8% were female. A literature review has revealed that men are at a higher risk of developing IHD than women and tend to develop it at a younger age with more severe symptoms and a higher mortality rate. Furthermore, women are likely to have a worse prognosis and poorer quality of life after an IHD diagnosis than men [25]. These gender differences in IHD prevalence, severity, and prognosis suggest that there may be differences in the underlying biological mechanisms of IHD between men and women. To address this, further research is needed to understand the underlying biological pathways of IHD to develop gender-specific treatments for this condition.

The study's limitations include a single study location, only one ethnic group included, and poor data documentation for some patients, particularly NYHA classification and underlying etiology of acute decompensation. Therefore, a multicenter and multiethnicity study is recommended to make more relevant comparisons.

## Conclusions

Our study aimed to report the demographics and characteristics of ADHF admitted to KAUH and analyze the gender discrepancies and rate of in-hospital mortality. We found more than half of our sample to be males, and diabetes mellitus and hypertension were common among ADHF patients. Elevated lab results at the time of presentation and patients with longer lengths of hospitalization were found to be associated with an increased risk of in-hospital mortality. This indicates the importance of understanding how such patients may present and how physicians could approach these cases. We recommend establishing education programs for patients on medication compliance. Furthermore, we strongly recommend conducting a multicenter and multiethnic study to explore the variability in patient presentations and management strategies.
